# Graphene Array-Based Anti-fouling Solar Vapour Gap Membrane Distillation with High Energy Efficiency

**DOI:** 10.1007/s40820-019-0281-1

**Published:** 2019-06-10

**Authors:** Biyao Gong, Huachao Yang, Shenghao Wu, Guoping Xiong, Jianhua Yan, Kefa Cen, Zheng Bo, Kostya Ostrikov

**Affiliations:** 1 State Key Laboratory of Clean Energy Utilization, College of Energy Engineering, Zhejiang University, Hangzhou, 310027 Zhejiang People’s Republic of China; 2 Department of Mechanical Engineering, University of Nevada, Reno, NV 89557 USA; 3 Joint CSIRO-QUT Sustainable Processes and Devices Laboratory, P.O. Box 218, Lindfield, NSW 2070 Australia; 4 School of Chemistry, Physics and Mechanical Engineering, Queensland University of Technology, Brisbane, QLD 4000 Australia

**Keywords:** Solar energy, Plasma-made nanostructures, Photothermal conversion, Water purification

## Abstract

Photothermal membrane distillation (MD) is a promising technology for desalination and water purification. However, solar-thermal conversion suffers from low energy efficiency (a typical solar-water efficiency of ~ 50%), while complex modifications are needed to reduce membrane fouling. Here, we demonstrate a new concept of solar vapour gap membrane distillation (SVGMD) synergistically combining self-guided water transport, localized heating, and separation of membrane from feed solution. A free-standing, multifunctional light absorber based on graphene array is custom-designed to locally heat the thin water layer transporting through graphene nanochannels. The as-generated vapour passes through a gap and condenses, while salt/contaminants are rejected before reaching the membrane. The high solar-water efficiency (73.4% at 1 sun), clean water collection ratio (82.3%), excellent anti-fouling performance, and stable permeate flux in continuous operation over 72 h are simultaneously achieved. Meanwhile, SVGMD inherits the advantage of MD in microorganism removal and water collection, enabling the solar-water efficiency 3.5 times higher compared to state-of-the-art solar vapour systems. A scaled system to treat oil/seawater mixtures under natural sunlight is developed with a purified water yield of 92.8 kg m^−2^ day^−1^. Our results can be applied for diverse mixed-phase feeds, leading to the next-generation solar-driven MD technology.

**Electronic supplementary material:**

The online version of this article (10.1007/s40820-019-0281-1) contains supplementary material, which is available to authorized users.

## Highlights

- New concept of solar vapour gap membrane distillation (SVGMD) is based on synergizing of nanochannel-guided water transport, localized heating, and membrane separation from feed solution.
- First-time introduction of the gap enables long-term stability and non-fouling membrane.
- SVGMD exhibits a solar-water energy efficiency higher than state-of-the-art solar vapour systems.

**Electronic supplementary material:**

The online version of this article (10.1007/s40820-019-0281-1) contains supplementary material, which is available to authorized users.

## Graphical abstract

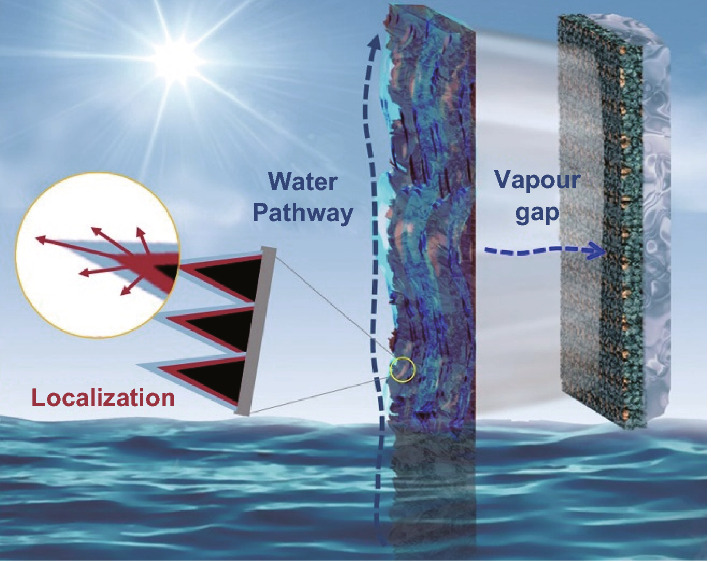

## Introduction

Solar energy with abundant availability has been widely utilized via photovoltaics, photocatalysis, and photothermal conversion [1, 2]. Solar desalination and water purification, heating saline/contaminated water for clean vapour generation by photothermal conversion, have attracted tremendous attention in addressing the pressing global issues of water scarcity and water contamination, but facing challenges to achieve efficient energy utilization [3, 4]. Recently, a new concept of heat localization was proposed for solar vapour generation with a high energy efficiency of 85% [5], in which heat dissipation was dramatically reduced by localizing heat at the interface between a light absorber and a thin water layer. Although subsequent works have been devoted to developing various light-absorbing materials [6–9], the performance of collecting clean water from the hot vapour, which is important for practical applications, still remains far below the expectations [7, 10]. Most of the previous works often employ a transparent plate/dome to collect the clean water, where the as-generated vapour is naturally condensed and then falls down under gravity force. According to the previous report [10], only 40% of clean water could be collected from the hot vapour by the natural condensation method, corresponding to a low solar-water energy efficiency of 22%. Moreover, the vapour mist and the water droplets condensing on the plate/dome would partially block the incident light by absorbing or reflecting, which also leads to a high optical energy loss up to 35% [10]. Therefore, significant improvements in the energy utilization efficiency are warranted, especially the clean water collection ratio and the solar-water energy efficiency.

Recently, photothermal membrane distillation (MD) [11–17], introducing polymer membrane into the solar vapour generation systems, has shown a potential to improve the water collection ratio and the solar-water energy efficiency. The membrane acts as a physical barrier to separate vapour from water (only vapour molecules are able to pass through the membrane) [18]. Meanwhile, the membrane can prevent most of the dissolved salt, organic micropollutants, and microorganisms from the feed side [19]. However, conventional photothermal MD systems face two persistent issues, i.e. insufficient energy conversion and membrane fouling.

Various light-absorbing nanomaterials (e.g. graphite oxide nanoflakes, carbon black nanoparticles, and Ag nanoparticles) have been proposed to improve the harvesting of solar energy [11–17]. However, the solar-water energy efficiency in conventional photothermal MD systems typically remains at a relatively low level of 45.0–53.8% [11–17], which could be attributed to two main reasons. First, conventional systems heat a large volume of feed water, which continuously dissipates heat to adjacent environment and leads to energy loss [5, 6]. Consequently, localized heating with directional heat transfer (i.e. from the light absorbers directly to just the thin water layer that needs to be heated) is thus highly desirable to increase the solar-water energy efficiency [5, 8, 9]. Second, the presence of light-absorbing nanomaterials on or inside the membrane often diminishes the benefits of otherwise better harvesting of solar energy. Indeed, light-absorbing materials (nanoparticles or nanoflakes) are densely distributed over or even incorporated into the membrane, e.g. through electrospinning, casting, and vacuum filtration [11, 13, 15]. Although higher loading of light-absorbing nanomaterials could improve light absorption [13, 20], it may block the membrane pores, leading to the higher vapour transport resistance and reduced solar-water energy efficiency [12, 13].

The second persistent issue is the membrane fouling during the membrane distillation operation. Salts and chemical/oil-based contaminants in the feed are easily deposited on the membrane surface, which leads to a deteriorated desalination/purification performance and even an irreversible membrane degradation [21, 22]. For example, after only 8-h operation, the fouling problem on a commercial polytetrafluoroethylene (PTFE) membrane leads to the severe decline of desalination performance [21, 23]. Major efforts were devoted to enhancing long-term process stability by modifying membranes using anodization, additional layers and coatings, etc. [21, 24–26]. Unfortunately, these techniques suffer from complex procedures and possible environmental pollution due to the use of toxic chemical reagents [21, 27]. Thus, new strategies are desirable to improve the energy efficiency and simultaneously address the membrane fouling issue.

**Fig. 1:**
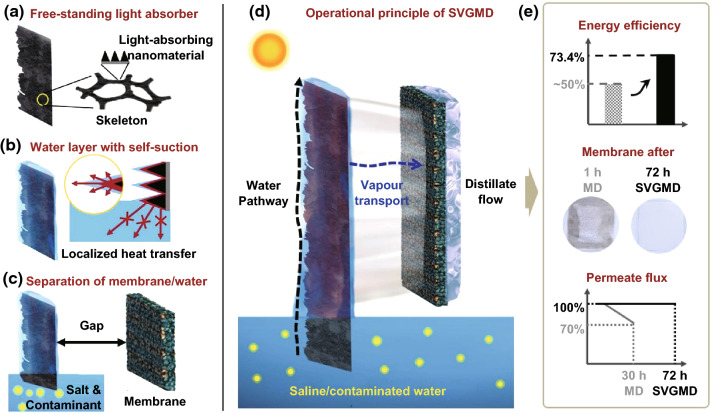
Solar vapour gap membrane distillation (SVGMD): enabling concept, key features, and operational principle. **a–c** Summary of the key features: **a** free-standing light absorber consisting interconnected skeleton and light-absorbing nanomaterials; **b** formation of thin water layer with self-suction and localized heating. **c** Solar vapour in the gap which separates the membrane from the water. **d** Schematic of SVGMD. Superhydrophilic light absorber ensures fast water suction. Under solar illumination, localized heat transfer leads to the efficient generation of solar vapour in the gap, which passes through the membrane and condenses with the distillate flow. **e** The SVGMD concept and scalable water purification system with high solar-water energy efficiency, excellent anti-fouling performance, and stable permeate flux

Herein, we demonstrate a new concept of solar vapour gap membrane distillation (SVGMD) synergistically combining self-guided water transport, localized heating, and separation of membrane from feed solution. It uses a free-standing, multifunctional light absorber (Fig. 1a), which enables a thin water layer on its surface with self-suction from the bulk feed solution to realize heat localization (Fig. 1b). Meanwhile, a deliberately introduced small ‘gap’ separates the membrane from the salt/contaminant solution, addressing the fouling issue (Fig. 1c). Under illumination, the thermal energy converted from solar light can be efficiently transferred to the thin water layer surrounding the light absorber, leading to a localized heat transfer and highly efficient generation of clean vapour (Fig. 1d). The vapour diffuses to the gap opposite to the incident light and collected by the distillate flow at the opposite side of the membrane, which overcomes the light blocking issue and simultaneously improves the water collection performance. In the current proof-of-concept work, a free-standing graphene–nickel foam with polymer coating is custom-designed via advanced plasma-based nanotechnology and used as the nanostructured light absorber (denoted as P–G–Ni_foam_). Taking the advantages of the unique design, very high solar-water energy efficiency of 73.4%, clean water collection ratio, excellent anti-fouling performance, and long-term stability over 72 h are simultaneously demonstrated in the SVGMD system (Fig. 1e). For scaled treatment of mineral oil/saline water mixture (1 g L^−1^ mineral oil with natural seawater), a daily purified water yield of 92.8 kg m^−2^ day^−1^ under natural sunlight is achieved.

## Experimental

### Fabrication of P–G–Ni_foam_

Commercial nickel foams with a thickness of 0.4 mm were cut into size of 4 × 10 cm^2^ and used as the substrate. Graphene array (i.e. the so-called vertically oriented graphenes, carbon/graphene nanosheets, carbon/graphene nanoflakes, graphene pedals, and carbon nanoflowers) is grown on nickel foam through plasma-enhanced chemical vapour deposition (PECVD) method [28]. After carefully cleaned, substrates were placed into a cylindrical quartz tube and heated to 700 °C under a base pressure below 3 Pa. The growth was conducted using CH_4_/H_2_ mixture (5 mL min^−1^: 5 mL min^−1^) as the precursor. During deposition, a radio frequency source (250 W) was coupled to the quartz tube and the pressure was maintained at approximately 100 Pa. After 1-h growth, the power was shut down and the sample was cooled down to room temperature under the protection of argon (10 mL min^−1^) [29].

PEDOT-PSS (poly(3,4-ethylenedioxythiophene)-poly(styrenesulfonate), 1.5 wt% dispersion in water) was purchased from Macklin Biochemical Co., Ltd, Shanghai, China. Before coating, the graphene–nickel foam was polished with acetone and methanol and then stored in dry atmosphere. The solution was sprayed onto the graphene–nickel foam with 0.2 MPa compressed air using a spray gun. After nitrogen annealing at 100 °C for 30 min, PEDOT-PSS layer was formed on its surface.

### Material Characterization

The morphology and microstructure of samples and membranes were characterized by scanning electron microscope–energy-dispersive X-ray spectrometry (SEM–EDX; SU-70). For light absorption tests, transmission (*T*) and reflection (*R*) were measured from 200 to 2600 nm using a spectrophotometer (UV-3150 UV–VIS), which enabled measurement across the ultraviolet, visible, and near-infrared regions. Absorption (*A*) can be calculated by *A* = 1 − *T* − *R*.

### Experimental Set-Up and Protocol

The laboratory-scale experiments were carried out with the self-made apparatus, which consists of three parts: (1) a plate has a quartz window with dimensions of 2 × 2 cm^2^ to allow sunlight illumination which a certain volume of water was stored in the tank below; (2) polypropylene spacers were placed in the middle of the membrane and P–G–Ni_foam_; (3) a plate has a small window with dimensions of 2 × 2 cm^2^ to allow vapour escape. Three parts were integrated with screws and nuts. Except for the quartz cover, all parts of the apparatus were well insulated thermally to make the heat loss as small as possible. The mass change was monitored using a precision microbalance (CPA225D, Sartorius) with an accuracy of 0.1 mg. A solar simulator (PLS-SXE300D, Beijing Perfect Light Technology Co., Ltd.) was used to generate light illumination which is equipped with a xenon lamp. The optical power was measured by an optical power meter (PD130, Beijing Perfect Light Technology Co., Ltd.).

In scaled SVGMD system, the quartz window is 2 mm in thickness with dimensions of 3 × 7 cm^2^. The flow channels (2 mm in height) of the feed and distillate were made in each of two blocks. A polypropylene spacer was placed in the middle of the membrane and P–G–Ni_foam_, and a plastic cross-mesh spacer was installed in the channel to support the membrane, while maintaining laminar flow. Both feed and distillate were concurrently circulated through the membrane module using two peristaltic pumps. The increase in the distillate reservoir was recorded by a precision balance (CPA225D, Sartorius). A 30 × 30 cm^2^ Fresnel lens was used to concentrate sunlight on the membrane surface by a factor of 12. The SVGMD module was enclosed in a box to prevent excess solar illuminating on the module.

All the measurements of the salinity and conductivity were taken using the conductivity meter (SX711, Shanghai Sanxin). TOC was measured using a TOC/TN analyser (TOC-VCSH, Shimadzu, Kyoto). The concentrations of five primary ions (Na^+^, K^+^, Mg^2+^, Ca^2+^, and B^3+^) in the natural seawater and the permeate water were measured by the inductively coupled plasma optical emission spectroscopy (ICP-OES, with an accuracy of 0.1 mg L^−1^). Size of the oil droplet in the mixture was analysed by dynamic light scattering (DLS) measurements (Zetasizer Nano ZS, Malvern Panalytical, UK). PVDF membrane used in this work was purchased from Millipore Corporation, 0.22 μm in pore size and 125 μm in thickness.

Control experiments were conducted using a direct contact membrane (PVDF) test apparatus. The flow channels (4 mm in height) of the feed and distillate were made in each of two blocks. The total active membrane area was 2 × 2 cm^2^. The temperature of inlet feed solution is 25 °C, while that of the distillate inlet stream is 20 °C.

### Estimation of Solar-Water Conversion

The solar-water energy efficiency is defined as the proportion of a given quantity of solar energy used for clean water production:

1 $$\eta_{\text{solar-water}} = \frac{{J \left( { h_{\text{lv}} + Q } \right)}}{{C_{\text{opt}} q_{\text{i}} }}$$

where *J* denotes the permeate flux across the membrane (*J* = *J*_light_ − *J*_dark_), *J*_light_ the permeate flux under illumination, *J*_dark_ the permeate flux at a dark environment, *h*_lv_ the phase change enthalpy (2256 kJ kg^−1^), *Q* the sensible heat [*Q* = *c* (*T*_v_ − *T*_l_)], in which *c* can be assumed as a constant (4.2 kJ kg^−1^ K^−1^), *T*_v_ is the temperature of vapour phase, and *T*_l_ is the initial temperature of water, *C*_opt_ is the optical concentration, and *q*_i_ is the nominal direct solar irradiation of 1 kW m^−2^.

## Results and Discussion

### Concept and Operation of SVGMD

Unlike conventional photothermal MD where the light-absorbing nanomaterials (e.g. nanoflakes and nanoparticles in the form of dispersion) are deposited onto the surface of membrane or even incorporated into the membrane [11–14], the key feature of SVGMD is the use of a graphene array-based free-standing, multifunctional light absorber separated from the membrane (Fig. 1a).

Beyond its intrinsic role of harvesting solar energy, the light absorber in the SVGMD system has the following features:

1. It is superhydrophilic and can pump water via graphene nanochannels from the bulk feed solution. A thin water layer is rapidly formed on the surface of the light absorber, and the thermal energy converted from the light illumination can be used to directionally heat the confined water layer, rather than heat the bulk solution (Fig. 1b). Such an excellent heat localization induced by self-guided water transport and engineered water pathways could significantly increase the solar-water energy efficiency.
2. It is standalone (i.e. not attached to the membrane) and thus enables a ‘gap’ between the light absorber and the membrane (Fig. 1c). Salt/contaminant-free vapour (instead of saline/contaminated water in common devices) contacts with the membrane and condenses on the distillate side. It could largely prevent the deposition of the salt/contaminant and thus resolve the fouling problem without any complex chemical modification on the membrane.
3. It is underwater superoleophobic and anti-salt-clogging, which could prevent the accumulation of the salt residues and weaken the adhesion of oil on its surface.

The operational principle of SVGMD is schematically shown in Fig. 1d. The bottom part of the light absorber is dipped into the bulk saline/contaminated feed. The feed water is lifted to the upper part of the light absorber while the oil is rejected underwater, which is attributed by the superhydrophilic and underwater superoleophobic nature of the light absorber. Under illumination, the light-absorbing materials of graphene arrays capture solar light and convert it into thermal energy. Through directional heat transfer, the thin layer of water surrounding the graphene arrays is heated. As a consequence, vapour is generated via liquid/vapour phase change and fills up the gap, leading to an increased vapour pressure. Driven by the pressure gradient between the two sides of membrane (serves as a physical barrier between the distillate flow and the water vapour), the hot vapour transports through the membrane and then condenses at the opposite side due to the cooling effect of distillate flow. Specifically, the membrane plays a key role in microorganism removal. For instance, bacteria could be rejected and absorbed by the membrane, as shown in Fig. S1. Meanwhile, the fouling problem of membrane caused by the direct contact with feed water in conventional MD systems [30] is rationally solved, dramatically improving the membrane lifespans and long-term stability. Detailed comparison between the current SVGMD and conventional photothermal MD systems in terms of system design, water flow, and heat transfer is given in Table S1.

### Efficient Solar-Thermal Conversion of P–G–Ni_foam_

An ideal light absorber of the SVGMD should be free-standing and simultaneously be able to absorb solar light, pump water from feed solution, and reject salt/contaminant (e.g. oil). In the current proof-of-concept work, the light absorber is produced by directly growing graphene arrays on the surface of nickel foam through a one-step PECVD process. The upper-layer graphene array serves as light-absorbing material to capture solar energy when the supporting nickel foam works as the skeleton to ensure its entirely standalone form and mechanical robustness. The as-synthesized hierarchical structure is then coated with PEDOT-PSS, which converts the wettability of graphene array from naturally hydrophobic to superhydrophilic and minimizes the accumulation of salt.

**Fig. 2:**
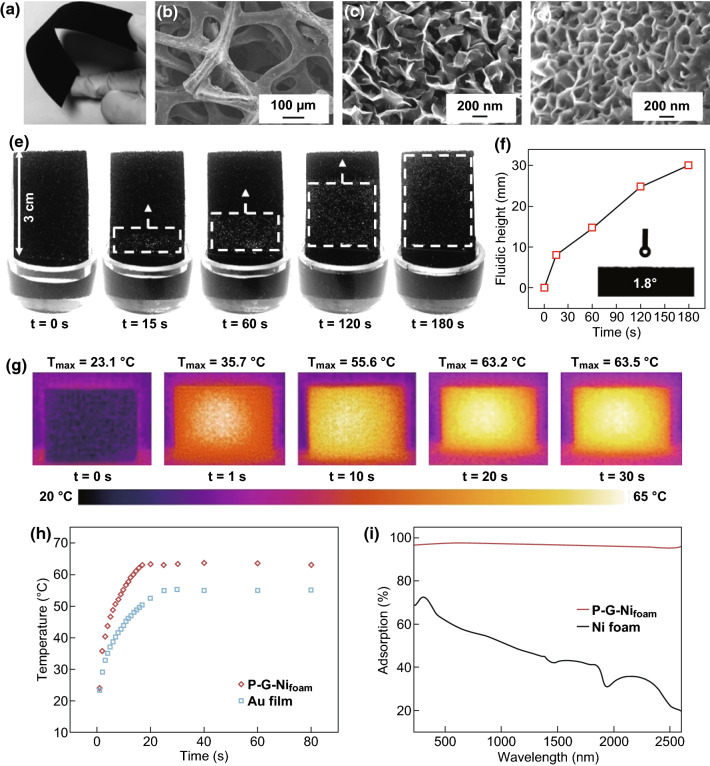
Free-standing, multifunctional P–G–Ni_foam_ layer for solar energy absorption, water transport, and solar vapour generation. **a** Optical image of the P–G–Ni_foam_. SEM image of **b** microporous structure of P–G–Ni_foam_ and **c**, **d** graphene array before and after PEDOT-PSS coating. **e** Fluidic transport across the P–G–Ni_foam_ in 180 s. **f** Flow height along with time. Inset: the static water contact angle measurement of P–G–Ni_foam_. **g** IR images of P–G–Ni_foam_ as a function of time at an illumination of 1 kW m^−2^. **h** Surface temperature of P–G–Ni_foam_ as a function of time at an illumination of 1 kW m^−2^. **i** Experimental absorption spectra of P–G–Ni_foam_ in the wavelength range of 200–2600 nm. All tests were performed at a room temperature and atmospheric pressure

Figure 2a shows a photograph of the light absorber, i.e. P–G–Ni_foam_, which exhibits a free-standing, flexible, and mechanically robust structure. SEM image of Fig. 2b shows its three-dimensional frameworks. Magnified SEM images in Fig. 2c, d show its upper-layer graphene array before and after PEDOT-PSS coating, respectively. A dense oriented plasma-made nanostructure is deposited on the nickel scaffold, and the span width of an individual graphene nanosheet is approximately 300 nm [31]. The pristine graphene array is naturally hydrophobic (water contact angle of 161.1°, see Fig. S2). After PEDOT-PSS coating, P–G–Ni_foam_ becomes superhydrophilic with a water contact angle of 1.8° (inset of Fig. 2f) and thus can effectively pump water from feed solution via graphene nanochannels. Figure 2e shows fluidic transport across the P–G–Ni_foam_ over 180 s. The height, width, and thickness of the sample are 4.5 cm, 2.0 cm, and 0.4 mm, respectively. The bottom region is dipped into the feed water. Due to its superhydrophilicity and the capillary forces induced between the neighbouring graphene nanosheets, the feed water is rapidly pumped and flows across the sample with a transport direction from the bottom to top. The flow rate within the first 15 s is 0.48 mm s^−1^, which is comparable with the performance of previously reported material with superior water transport behaviour [32]. Meanwhile, the P–G–Ni_foam_ light absorber exhibits an underwater superoleophobicity (characterized by the oil contact angle of 151.8°, see Fig. S3), making it possible to repel oil from its surface underwater.

The solar-thermal conversion performance of P–G–Ni_foam_ is characterized with an infrared (IR) camera. The sample is exposed to a constant illumination of 1 kW m^−2^ (i.e. 1 sun), and the IR images at different exposure times are recorded. As shown in Fig. 2g, the surface temperature of P–G–Ni_foam_ rapidly increases from 23.0 to 35.7 °C within 1 s and eventually reaches a steady state (63.2 °C) at 20 s. Figure 2h shows the surface temperature of P–G–Ni_foam_ (at central region) as a function of time at an illumination of 1 kW m^−2^. For comparison, the temperature increase at the surface of a 60-nm-thick Au film is measured. While Au is commonly considered as an excellent photothermal material [9], P–G–Ni_foam_ presented faster temperature increase response and higher stable temperature. The effective solar-thermal conversion and fast self-heating of P–G–Ni_foam_ could be mainly attributed to its superior photonic absorption. Figure 2i shows the absorption spectra of P–G–Ni_foam_ with a broad wavelength ranging from 220 to 2600 nm. The average light absorption of P–G–Ni_foam_ is calculated as 97.1%, which is much higher than that of the pristine nickel foam (43.1%). Meanwhile, its three-dimensional, interconnected skeleton with the decorated graphene open nanochannels also favours light absorption. The light reaching the surface of P–G–Ni_foam_ will be trapped in the dense graphene arrays and then almost completely absorbed after multiple internal reflections in graphene nanochannels (schematically shown in Fig. S4), instead of reflection or transmission, resulting in the broadband and highly efficient solar light absorption [33].

### High Solar-Water Energy Efficiency of SVGMD

**Fig. 3:**
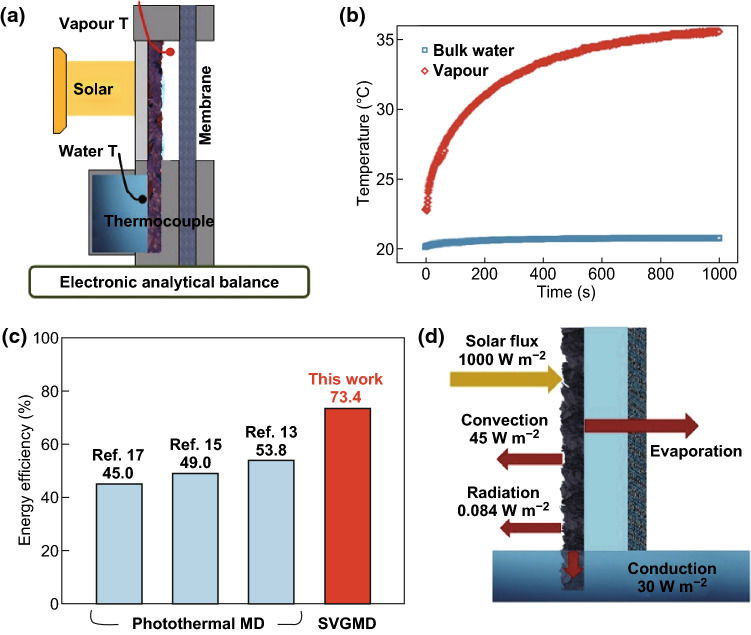
Record-high solar-water efficiency in SVGMD and its excellent performance in desalination of natural seawater and salty brine and purification of mineral oil-contaminated water. **a** Schematic of SVGMD with the solar simulator. **b** The temperature of vapour in the gap and bulk water in the tank as a function of time at an illumination of 1 kW m^−2^. **c** Comparison of energy efficiency between current work and previous studies [13, 15, 17]. **d** The energy balance and heat transfer in SVGMD

The P–G–Ni_foam_ light absorber is then applied to the SVGMD system for solar desalination and purification. The experimental system is schematically shown in Fig. 3a. The solar simulator with a xenon lamp is equipped with an optical filter to obtain the standard AM 1.5G solar spectrum. The gap width between P–G–Ni_foam_ and a commercial polyvinylidene fluoride (PVDF) membrane is set as 1 mm, which is optimized through a series of tests (see Fig. S5). Natural seawater from Hainan Island (110.1°E, 20.0°N) is used as the feed solution. An illumination of 1 sun is employed to simulate the solar radiation. Thermocouples are used to measure the temperature evolutions of the hot vapour in the gap and the bulk liquid in the tank. As shown in Fig. 3b, the vapour temperature gradually increases from 22.4 to 36.1 °C. In contrast, the bulk water has a negligible temperature rise less than 1 °C. It suggests that the as-obtained thermal energy is effectively confined in the thin water layer only existing in graphene nanochannels, leading to a localized heat transfer and minimal heat dissipation.

Although materials with excellent solar harvesting ability (e.g. polydopamine, light absorption coefficient of 97.0%) [17] and good water permeability/wettability (e.g. mixture of polydopamine and reduced graphene oxide, water contact angle of 46.0°) [15] have been used, the solar-water energy efficiency of conventional photothermal MD devices still remains below 50%. Even at an elevated ambient temperature of 40 °C, the solar-water energy efficiency of conventional photothermal MD is still low (53.8%) [13]. At the present SVGMD, a high permeate flux of 1.10 kg m^−2^ h^−1^ is obtained at 1 sun. Correspondingly, the solar-water energy efficiency is calculated to be 73.4%, significantly outperforming the previous works (45.0–53.8%) [13, 15, 17], as shown in Fig. 3c. Additionally, the solar-water efficiencies can be further improved by increasing the solar density to 5 sun and 10 sun, whose details are available in Fig. S6.

The superior solar-water efficiency of the current SVGMD could be predominantly attributed to the following three factors. First, the free-standing P–G–Ni_foam_ presents broadband and highly efficient photonic absorption (i.e. 97.1% at the wavelengths ranging from 220 to 2600 nm), exhibiting superior ability to harvest solar energy. Second, the localized heating achieved by engineered water pathways dramatically suppresses the heat loss to bulk liquid and leads to directional heat transfer from light absorber to the thin water layer. Third, the graphene nanosheets with dense, sharp edges can serve as nanoscale fin-like heat exchangers, leading to highly effective directional heat transfer and very low heat losses. Thermal analysis on the current SVGMD system is conducted (see details in Section S8), and the results are presented in Fig. 3d. The convection and radiation heat loss to the adjacent environment and the conductive heat loss to bulk water are calculated as 45, 0.08, and 55 W m^−2^, respectively, while the total heat loss is only ~ 10% of the incident solar energy (1000 W m^−2^).

### Long-Term, Effective Desalination and Purification

To demonstrate the feasibility of SVGMD to treat diverse solutions, natural seawater, highly saline brine and oil-contaminated water have been used as the feed. Two main applications are demonstrated as follows.

**Fig. 4:**
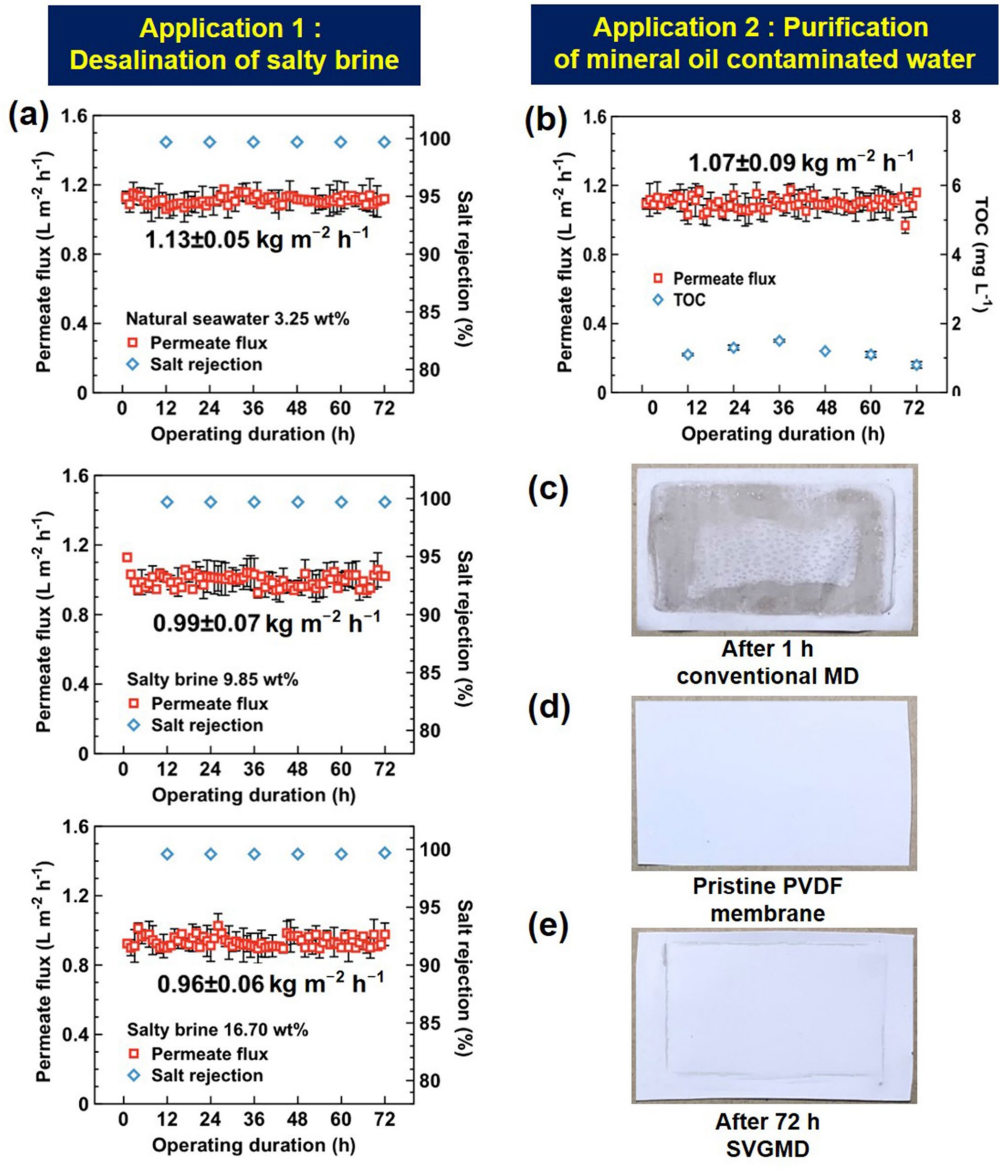
Two main applications for long-term, effective desalination and purification. **a** The permeate flux and salt rejection of SVGMD for natural seawater (3.25 wt%) and two kinds of salty brine (9.85 and 16.70 wt%) at an illumination of 1 kW m^−2^. **b** The permeate flux and TOC level of purified water of SVGMD when processing mineral oil-contaminated water at an illumination of 1 kW m^−2^. **c**–**e** Optical images of PVDF membrane **c**, after processing mineral oil-contaminated water in the conventional MD over 1 h (**d**) and in the SVGMD system over 72 h (**e**). The error bar comes from three repeated tests. All tests were performed at a room temperature and atmospheric pressure

The first application is to process natural seawater (3.25 wt%) and two types of salty brine (9.85 and 16.70 wt% of NaCl solution). Figure 4a shows the permeate flux of distilled water and salt rejection rate over 72 h of consecutive operation at 1 sun. For all the tests, the salt rejection rates (see Section S9 for calculation method) are close to 100%. Even for salinity as high as 16.70 wt%, the salt rejection rate is at a high level of > 99.6%. To evaluate the long-term operation stability, the permeate flux of distilled water is recorded every 1 h. For three types of saline brine (natural seawater, 9.85 and 16.70 wt% of NaCl solution), the average permeate flux over 72 h is 1.13 ± 0.05, 0.99 ± 0.07, and 0.96 ± 0.06 kg m^−2^ h^−1^, respectively. The very small fluctuation of both the salt rejection rate and the permeate flux of distilled water confirms the excellent long-term stability of the current SVGMD system. SEM and EDX analyses (see Section S10) show no noticeable salt deposition on the membrane after 72 h of SVGMD operation, which may dramatically expand the membrane’s lifespans.

The second application is to process mineral oil-contaminated water (i.e. 1 g L^−1^ mineral oil and 1 mM NaHCO_3_ in water). Figure 4b shows the permeate flux and total organic carbon (TOC) level of purified water after 72-h consecutive test at an illumination of 1 kW m^−2^. The TOC concentration in the distillate is at a low level of < 2 mg L^−1^, clearly better than the World Health Organization (WHO) standard for drinking water (5 mg L^−1^) [34, 35]. Meanwhile, stable permeate flux of 1.07 ± 0.09 kg m^−2^ h^−1^ is achieved. Liquids with low surface tension contaminants (e.g. surfactants and mineral oil) commonly cause detrimental membrane fouling, resulting in failure of conventional MD processes [21, 24, 25]. Control experiments using conventional MD are thus conducted (see “Experimental” section for details). As shown in Fig. 4c, d, oil-based contaminants are observed on the surface of the PVDF membrane after only 1-h operation of the conventional MD device. In contrast, as shown in Fig. 4e, the membrane used in the current SVGMD system remains clean after 72 h of operation. Wettability tests on the membrane before and after distillation are conducted, and the small change in the water contact angle further confirms that there is no obvious chemical/morphological change or oil-based contaminant residues on the membrane (see Fig. S9).

Different from the existing approaches based on complex modifications of commercial membranes [24, 25, 30, 36, 37], SVGMD can realize excellent anti-fouling performance using pristine membranes. The rationally designed gap effectively avoids direct contact between the saline/contaminated feed and the membrane, and thus, only clean vapour reaches the membrane. Instead, the free-standing P–G–Ni_foam_ layer contacts with the saline/contaminated water which is guided and confined over the superhydrophilic solid–liquid interface [38, 39]. Meanwhile, its underwater superoleophobic surface repels oil from the P–G–Ni_foam_ and its superhydrophilic nature avoids salt accumulation at the surface though ion diffusion and advection. Moreover, the PEDOT-PSS coating further prevents the adhesion of salt particles to the surface [40, 41]. Only a very few salt residues are observed on the surface of P–G–Ni_foam_ after long-term, consecutive distillation processes (see Fig. S10).

### Highly Efficient Water Collection

The performance of collecting clean water from the hot vapour is important for practical applications [14, 15]. In most of previous works, the solar-vapour efficiency is used to evaluate the solar desalination/purification performance, i.e. the proportion of a given quantity of solar energy used for vapour production at open environment, and is calculated as Eq. 2:

2 $$\eta_{\text{solar-vapour}} = \frac{{\dot{m} \left( { h_{\text{lv}} + Q } \right)}}{{C_{\text{opt}} q_{\text{i}} }}$$

where $\dot{m}$ denotes the vapour flux (kg m^−2^ h^−1^), calculated based on the mass decrease in the feed water. *h*_lv_ is the temperature-dependent latent heat of vaporization of water. *Q* is the sensible heat. *C*_opt_ is the optical concentration and *q*_i_ is the nominal direct solar irradiation of 1 kW m^−2^.

However, a key factor of water collection ratio is largely ignored. We reiterate that the water collection ratio, one of the key energy and matter efficiency indicators, has not recently attracted the attention. In typical solar desalination/purification systems, the vapour naturally condenses on a transparent plate/dome and then falls down into a container by gravitational force [10, 42]. Although high vapour generation rates and high solar-vapour efficiency up to 95% have previously been reported [5, 6, 8, 33, 42], the water collection ratio still remains at a low level (Only 40% of clean water could be collected from the hot vapour). As a result, the solar-water efficiency is only 22% [10], which means a large portion of the as-generated vapour needs to be further recycled, thus requiring additional energy input.

In addition, in conventional solar vapour systems, the incident light might be partially blocked (absorbed or reflected) by the vapour mist and the droplets forming on the plate/dome, as schematically shown in Fig. S11. The blocked light will also lead to an unwanted energy waste and diminish the vapour generation performance. In the current SVGMD system, the generated vapour diffuses to the gap at the side opposite to the incident light instead of the space between light source and absorber. Thus, the energy loss caused by vapour mist and condensed droplets is dramatically reduced. In addition, the cold distillate flow (6 mL min^−1^; 18.1 °C) at the opposite side of the membrane can facilitate the hot vapour to directionally transport through the membrane and condensed with the cold distillate flow.

The SVGMD thus inherits the advantage of MD in vapour condensation and water collection and meanwhile dramatically improves the energy utilizing efficiency. As shown in Fig. S11, a test to compare the performances of conventional solar vapour system with a dome and the current SVGMD is conducted. With the current P–G–Ni_foam_ absorber, the evaporation rate and solar-vapour efficiency from open environment can reach 1.41 kg m^−2^ h^−1^ and 90%, respectively. However, only 0.75 kg m^−2^ h^−1^ of clean water is collected and a low solar-water efficiency of 48.4% is obtained from the dome. On the other hand, the clean water collection rate in the current SVGMD can reach 1.16 kg m^−2^ h^−1^, which means 82.3% of vapour is recycled. Consequently, the high solar-water efficiency of 74.5% is obtained, which is 3.5 times higher compared to the existing solar vapour systems [10] and also much higher than the previous photothermal MD systems (45.0–53.8%).

### Scaled SVGMD Module for Clean Water Production

**Fig. 5:**
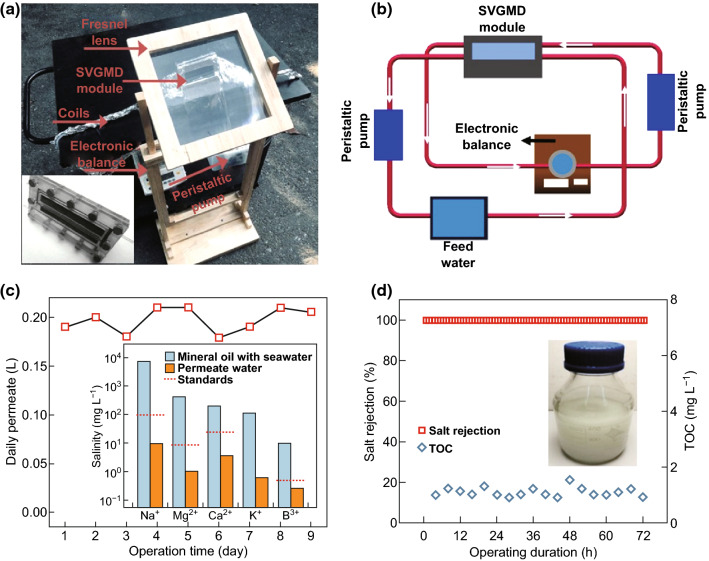
Scaled SVGMD module demonstrates excellent performance in desalination of mineral oil/seawater mixtures under real-world conditions. **a** Scaled SVGMD system with different components of the system marked with arrows. **b** Schematic of the SVGMD system. For experiments with solar concentration, a 30 × 30 cm^2^ Fresnel lens is used to concentrate sunlight on the P–G–Ni_foam_ surface by a factor of 12. **c** The daily permeate water of outdoor solar experiments with 12 × solar magnification. The inset is the concentrations of five primary ions before (mineral oil/seawater mixtures) and after (permeate water) desalination. **d** Salt rejection performance and TOC level of permeate water with seawater mixtures/mineral oil (1 g L^−1^ mineral oil with natural seawater). The inset shows a typical mineral oil/seawater mixture

To further demonstrate the applicability of SVGMD in practical conditions, an outdoor test is conducted with a scaled module operated in a closed loop configuration (Fig. 5a, b). A light exposure window of 3 × 7 cm^2^ and a Fresnel lens with 12-fold concentration are assembled to collect and concentrate solar energy. A mixture composed of natural seawater (3.25 wt%), 1 g L^−1^ mineral oil, and 1 mM NaHCO_3_ is applied as the feed solution with a flow rate of 2 mL min^−1^ (see section S14 for the oil size distribution of the emulsion mixtures and the composition of natural seawater). The opposing cold permeate side is cooled by a water flow of 6 mL min^−1^. The feed and permeate flows are continuously circulated using peristaltic pumps, which used very low (5 W) electric power from the grid and can easily be replaced by renewable energy (e.g. photovoltaic panels) in the portable, off-grid module. The scaled SVGMD module was operated for 9 days with a duration of 8 h per day (from 9:00 am to 16:00 pm) at Hangzhou city (120.1°E, 30.3°N), where the solar intensity ranged from 0.6 to 1.2 kW m^−2^ (see Fig. S13). As shown in Fig. 5c, the daily yield of clean water is approximately 0.195 kg day^−1^. Given the sample area of 21 cm^2^, the daily yield of fresh water is approximately 92.8 kg m^−2^ day^−1^ under natural sunlight. The salinity and the TOC in the collected water were monitored. After 9 days of operation, the scaled SVGMD module still maintains a high salt rejection close to 100% (see red squares in Fig. 5d). The ion concentrations of Na^+^, Mg^2+^, Ca^2+^, K^+^, and B^3+^ are reduced from 7328, 434, 202, 112, and 9.5 mg L^−1^ to 9.70, 1.04, 3.61, 0.62, and 0.26 mg L^−1^, respectively (inset in Fig. 5c), much better than the baseline levels of drinking water defined by WHO [34, 35]. The low TOC level (< 2 mg L^−1^) in purified water suggests that oil is repelled during the distillation process (see blue hexagons in Fig. 5d). The outcomes of this work demonstrate the potential of SVGMD for long-term, effective water purification with excellent simultaneous salt and oil rejection and point to SVGMD as a promising solution for remote water treatment or desalination in a household- or community-scale application.

## Conclusions

We demonstrate a new concept of SVGMD achieving a record-high solar-water energy efficiency and excellent anti-fouling property with long-term stability. The key feature of SVGMD compared with conventional phtotothermal MD systems is its free-standing, multifunctional light absorber, which simultaneously enables efficient light absorption, rapid suction of water, localized heating, and, importantly, directional transport of vapour into a small gap that separates the membrane from the saline/contaminated water. In the current proof-of-concept work, graphene array-based light absorber is fabricated by plasma-enhanced nanotechnology. With the synergistically combined advantages of heat localization and photothermal MD, the SVGMD system demonstrates a very high energy efficiency of 73.4% at 1 sun [11–17]. According to energy balance and thermal analysis on the SVGMD system, the total heat loss (including the convection and radiation heat loss to the adjacent environment, and the conductive heat loss to bulk water) is only~ 10% of the incident energy. Meanwhile, it inherits the advantage of MD in vapour condensation and water collection, enabling a high solar-water energy efficiency, 3.5 times higher than in the existing solar vapour systems. Further development is possible with the optimization of light-absorbing materials and water transport pathways.

Furthermore, the feasibility of the SVGMD system to treat diverse contaminant mixtures (e.g. with different and high levels of salinity and organic contamination) is demonstrated without membrane fouling that plagues many common methods. Such an excellent anti-fouling performance thus leads to the stable permeate flux over 72 h of consecutive operation and the long lifespans of the commercial membrane. Practical desalination of mineral oil/saline water mixtures in the scaled SVGMD module results in a daily purified water yield of approximately 92.8 kg m^−2^ day^−1^ under natural sunlight, indicating an excellent performance in vapour generation, condensation, and water collection. Our work therefore redefined the roles of the solar absorber and the membrane, which are different with conventional and photothermal MD systems. Based on the SVGMD concept and the outcomes of this work, our research could lead to next-generation solar harvesting-enabled energy-efficient membrane distillation technology for diverse water treatment applications that rely on simple membranes made of cheaper materials and last longer without cleaning.

## Electronic supplementary material

Below is the link to the electronic supplementary material.

Supplementary material 1 (PDF 1323 kb)
