# Supplementary material for: Graphene Array-Based Anti-fouling Solar Vapour Gap Membrane Distillation with High Energy Efficiency
Source: Nanomicro Lett. 2019 Jun 10;11:51. doi: 10.1007/s40820-019-0281-1 (PMC7770882; doi:10.1007/s40820-019-0281-1)
Supplement: Supplementary file 1 — Supplementary material 1 (PDF 1323 kb) [file 40820_2019_281_MOESM1_ESM.pdf]

Supporting Information for

Graphene Array based Anti-fouling Solar Vapour Gap Membrane Distillation with High Energy Efficiency

Biyao Gong[1], Huachao Yang[1], Shenghao Wu[1], Guoping Xiong[2], Jianhua Yan[1], Kefa Cen[1], Zheng Bo[1, *], Kostya (Ken) Ostrikov[3, 4]

[1]State Key Laboratory of Clean Energy Utilization, College of Energy Engineering, Zhejiang University, Hangzhou, Zhejiang 310027, People's Republic of China

[2]Department of Mechanical Engineering, University of Nevada, Reno, Nevada 89557, USA

[3]Joint CSIRO-QUT Sustainable Processes and Devices Laboratory, P.O. Box 218, Lindfield, NSW 2070, Australia

[4]School of Chemistry, Physics and Mechanical Engineering, Queensland University of Technology, Brisbane, Queensland 4000, Australia

Biyao Gong, Huachao Yang, and Shenghao Wu contributed equally to this work

*Corresponding author. E-mail: bozh@zju.edu.cn (Bo Zheng)

Supplementary Figures, Table and Discussion

S1 Microorganisms-removal Experiments

The microorganisms-removal experiments of SVGMD and conventional photothermal steam generation system are conducted using the standard commercial bio-indicator. The experimental setups are shown in Fig. S1. The bacteria carrier (thermophilic bacillus) is placed into the feed water. Then the purified water is transferred into the bio-indicator and incubated at 56 °C for 48 h. The color of bio-indicator after incubation is applied to indicate the microorganisms-removal results, in which the purple color indicates that the microorganisms can be completely rejected through the membrane and the spore survival does not occur, while the yellow color indicates the spore survival. As shown in Fig. S1, the bio-indicator containing the purified water from conventional photothermal steam generation system changes its color from purple to yellow. Conversely, the bio-indicator containing the purified water after SVGMD process has no change in color (yellow), revealing the microorganisms-removal performance of the membrane.

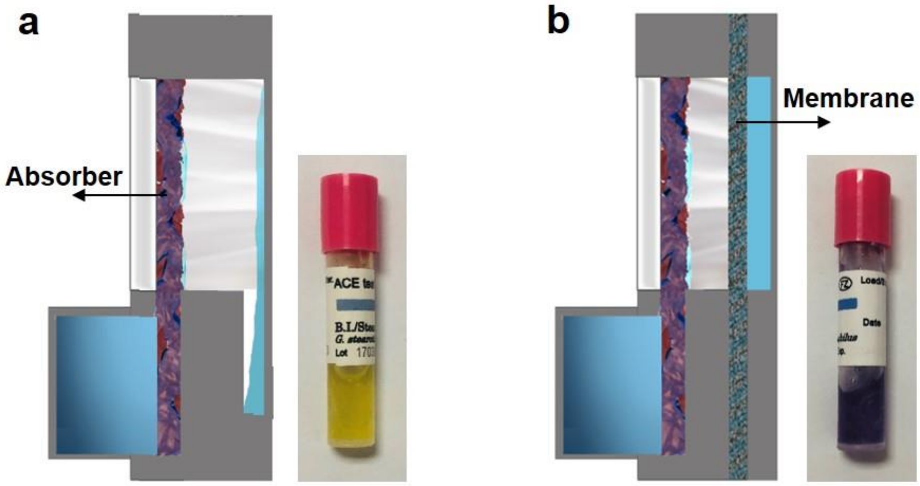

Fig. S1 **a** Schematic of the conventional photothermal steam generation system. The standard commercial bio-indicator changes its color from purple to yellow. **b** Schematic of the SVGMD system. The standard commercial bio-indicator has no change in color, indicating the microorganisms-removal performance. All tests were performed at an illumination of 1 kW m^{-2}.

S2 Issues in Conventional Photothermal MD and Proposed Concept to Solve These Issues

Table S1 A comparison between SVGMD and conventional photothermal MD in terms of system design, water flow, and heat transfer

	Photothermal MD	SVGMD
Structure	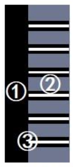 ① Light absorber<br>② Membrane<br>③ Coating (blocked pores)	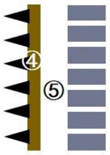 ④ Free-standing light absorber<br>⑤ Vapour gap
Water Flow	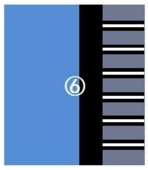 ⑥ Direct contact between membrane and water	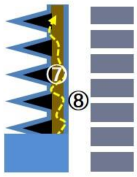 ⑦ Self-guided water transport<br>⑧ Separation of membrane from water
Heat transfer	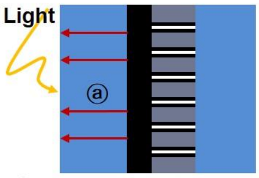 ⓐ Heat dissipation through feed water	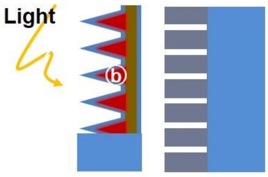 ⓑ Localized & directional heat transfer

Top-left: In conventional photothermal MD, the dispersion of light-absorbing materials (e.g., nanoflakes and nanoparticles) is deposited onto the surface of membrane or even incorporated into the membrane. It may block the membrane pores, leading to higher vapour transport resistance.

Middle-left: In conventional photothermal MD, the feed saline/contaminated water directly contacts with the membrane, leading to the contaminant deposition and thus the fouling problem.

Bottom-left: In conventional photothermal MD, the feed water flows through the photothermal membrane. The heat concentrated in the light absorber is transferred to the bulk feed water. Substantial amount of heat is lost to the bulk water.

Top-right: Our new conceptual design of free-standing absorber, which enables a gap between the membrane and the absorber. Graphene array is seamlessly anchored on the surface of robust three-dimensional nickel foam.

Middle-right: Water is transported by the free-standing light absorber, achieving the uniquely engineered water pathways. A thin water layer with self-guided transport from the bulk feed solution surrounds the absorber (i.e., graphene array). The feed saline/contaminated water no longer directly contacts with the membrane, thus resolving the fouling problem.

Bottom-right: With the engineered water pathways, the as-generated thermal energy is effectively confined in the thin water layer surrounding graphene nanosheets, leading to the surpressed heat loss to bulk water beneath and the directional heat transfer from the light absorber to the thin water layer.

S3 The Fabrication Procedure and Material Characterization of P-G-Ni$_{foam}$

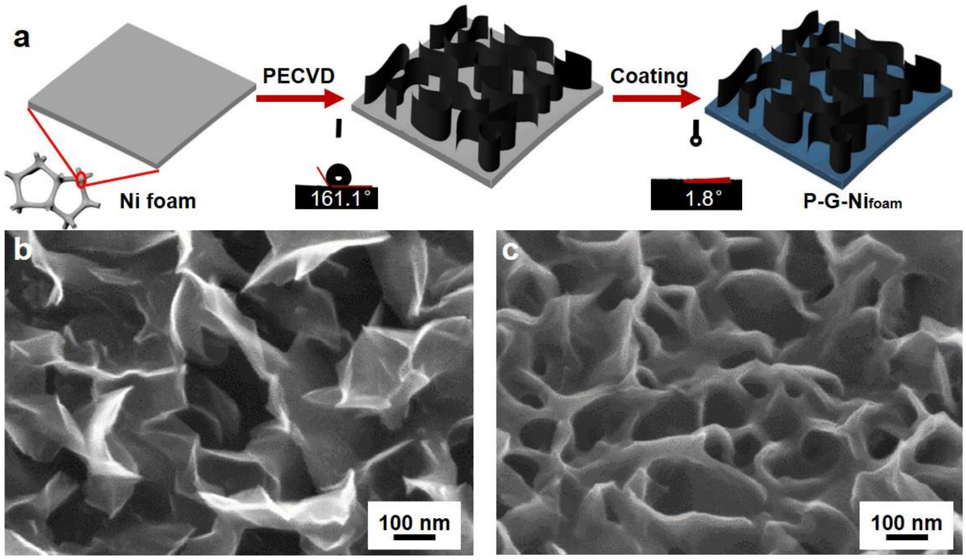

Fig. S2 a Schematic illustration of preparation of P-G-Ni$_{foam}$ via plasma-enhanced chemical vapor deposition and coating. P-G-Ni$_{foam}$ becomes superhydrophilic (water contact angle θ = 1.8°) after PEDOT-PSS coating. SEM images of P-G-Ni$_{foam}$ **b** before and **c** after PEDOT-PSS coating

S4 Underwater Oil Contact Angle Measurements of P-G-Ni$_{foam}$

The underwater oil contact angle was recorded using a computerized contact angle analyzer (DropMeter A-200, MAIST) and a high-speed camera (MotionXtra HG-100K, REDLAKE). Motor oil droplets (5 μL) were injected onto the surface using a motor-driven syringe device. Contact angles were calculated by the Young-Laplace equation.

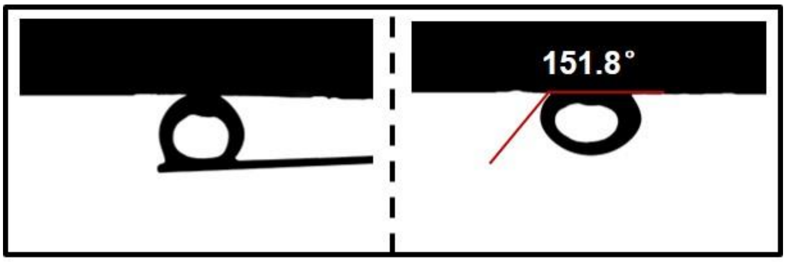

Fig. S3 Underwater oil contact angle measurements of P-G-Ni$_{foam}$. Left: Measurement of underwater oil contact angle. Right: Optical image of underwater motor oil droplets on P-G-Ni$_{foam}$. The underwater oil contact angle is 151.8°

S5 Excellent Light Harvesting Ability of P-G-Ni$_{foam}$

As shown in Fig. S4, the graphene array can trap incident light within the nanochannels instead of bouncing off or penetrating the absorber, thereby effectively suppressing the light transmission and reflection and maximizing the light absorption capability.

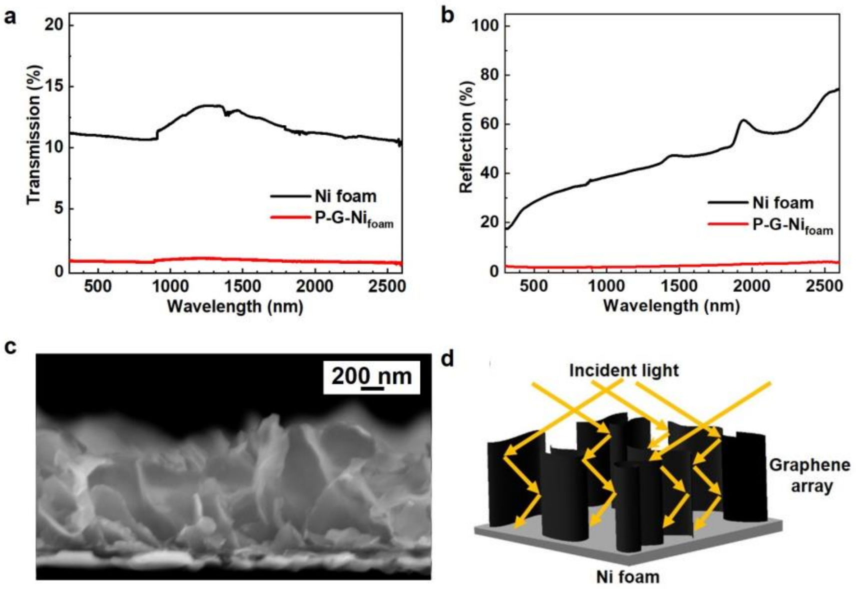

Fig. S4 Schematic of light reflection interactions with graphene array. **a, b** Photonic transmission **a** and reflection **b** spectra of Ni foam and P-G-Ni$_{foam}$. **c** Cross-sectional SEM image of P-G-Ni$_{foam}$. **d** Schematic of light undergoing multiple internal reflections through the oriented graphene array

S6 Influence of Gap Width on Solar Thermal Conversion

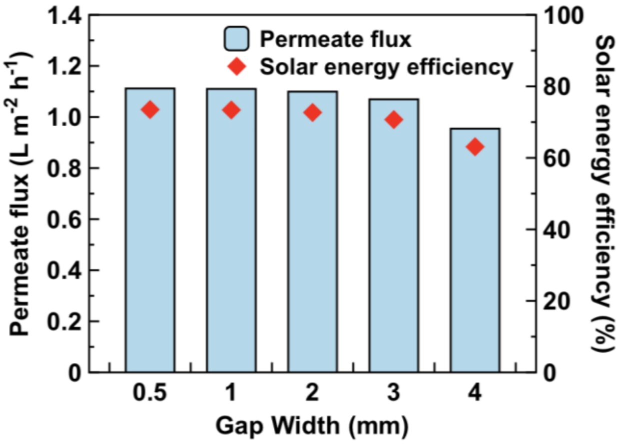

Fig. S5 The effect of the vapour gap width on flux and solar-water energy efficiency. The increase of the permeate flux is negligible as the gap width is reduced from 2 to 0.5 mm. So we choose a gap width of 1 mm. All tests were performed at room temperature and atmospheric pressure.

S7 High Solar-Water Energy Efficiency Under Different Light Intensities

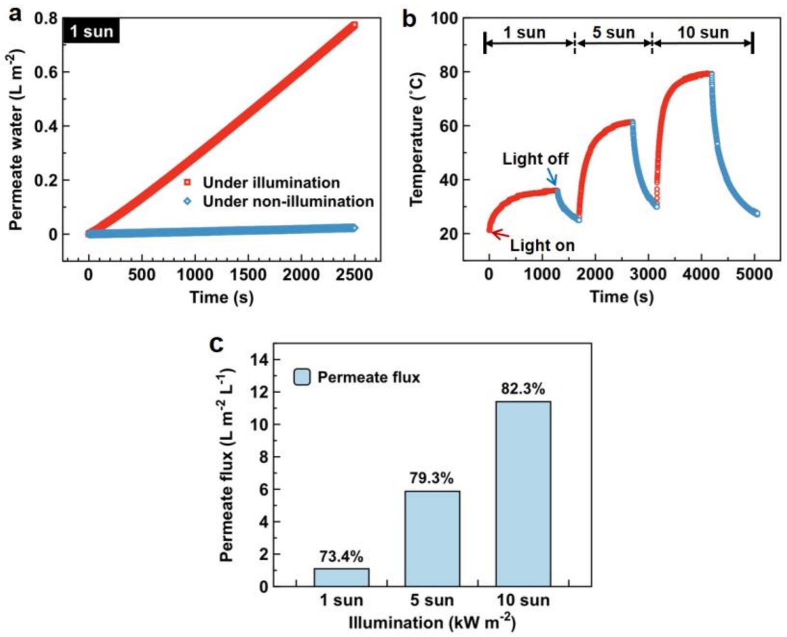

Fig. S6 The permeate flux and solar-water energy efficiencies at 5 sun and 10 sun of the SVGMD system. **a** Permeate water obtained at an illumination of 1 kW m^{-2} and under the dark environment. The permeate flux under the dark environment is subtracted from all the measured data under the solar illumination. **b** The vapour temperature as a function of time at an illumination of 1, 5, and 10 kW m^{-2}. **c** The permeate flux and solar-water energy efficiencies of the SVGMD system at an illumination of 1, 5 and 10 kW m^{-2}. All the tests were performed at a room temperature and atmospheric pressure.

Figure S6 shows the performance of SVGMD at 5 sun and 10 sun. Vapour temperature increases obviously with the illumination density, from 36.1 °C at 1 sun to 79.9 °C at 10 sun. Driven by the larger temperature gradient across the membrane, the permeate flux is prominently exaggerated by 10 folds, which are calculated to be 1.1, 5.9, and 11.4 L m^{-2} h^{-1} at 1, 5, and 10 sun, respectively. The corresponding energy efficiency is 73.4%, 79.3%, and 82.3%, respectively.

S8 Thermal Analysis

Thermal analysis of the solar-vapour conversion process has been conducted, including radiative (q_{rad}), convective (q_{conv}) heat loss to the surroundings and conductive heat loss to bulk water (q_{cond}). The energy dynamic equilibriumcan be expressed as Eq. S1

$$J\,(h_{lv}+Q)=\eta_{solar-vapor}q_{solar}=\alpha q_{solar}-q_{rad}-q_{conv}-q_{cond}$$

$$=\alpha q_{solar}-\varepsilon\sigma(T^4-T_\infty^4)-h(T-T_\infty)-\frac{k(T_{l1}-T_{l2})}{\Delta l} \qquad \text{(S1)}$$

Where α and q_{solar} denote the light absorption of the absorber and solar flux, respectively. The heat flux terms in Eq. S1 are as follows.

The radiative heat loss (q_{rad}) to the adjacent environment can be calculated as Eq. S2:

$$q_{rad}=\varepsilon\,\sigma\left(T_a{}^4-T_\infty^4\right) \qquad \text{(S2)}$$

Where ε is the emission of the absorber, σ is the Stefan-Boltzmann constant. T_a is the wet surface temperature of the absorber. T_∞ is the ambient temperature [S1, S2]

The conductive heat loss (q_{cond}) to bulk water can be calculated through the temperature gradient in the underlying water:

$$q_{cond}=\frac{k\,(T_{l1}-T_{l2})}{\Delta l} \qquad \text{(S3)}$$

Where k is the thermal conductivity of feed water. The temperature gradient in the underlying water below the sample is measured by two embedded thermocouples (i.e., T_{l1} and T_{l2}) placed in the water tank. The distance between the thermocouples Δl is 10 mm.

The convective heat loss (q_{conv}) to the adjacent environment can be calculated as Eq. S4:

$$q_{conv} = A\,h\,(T_a - T_\infty) \qquad \text{(S4)}$$

Where h is the convection heat transfer coefficient and is assumed to be 5 W m^{-2} K^{-1}.

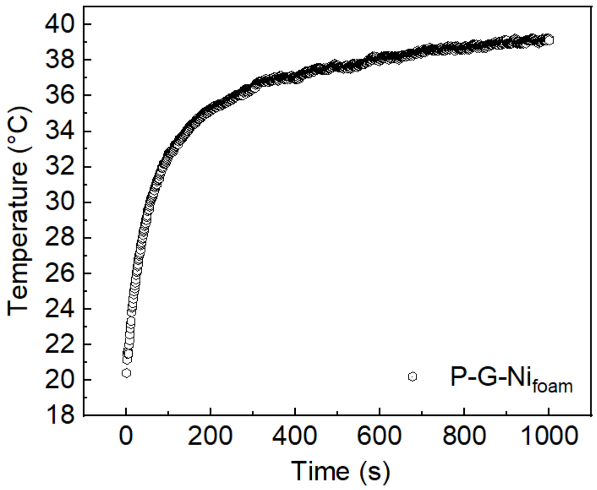

Fig. S7 The wet surface temperature of P-G-Ni_{foam} as a function of time in the SVGMD system. The temperature is stabilized at about 39.1 °C at an illumination of 1 kW m^{-2}, which could be approximated as the wet surface temperature of the absorber (T_α)

S9 Salt Rejection in the Current SVGMD System

Salt rejection is defined as a measure of the quality of the flux and the percent reduction in salinity after distillation process, which can be calculated as Eq. S5:

$$\text{Salt Rejection (\%)} = \left(\frac{\sigma_{feed} - \sigma_{distillate}}{\sigma_{feed}}\right) \qquad \text{(S5)}$$

where σ_{feed} is the conductivity of the feed, $\sigma_{distillate}$ is the conductivity of the distillate.

S10 SEM–EDX Analysis for the Membranes

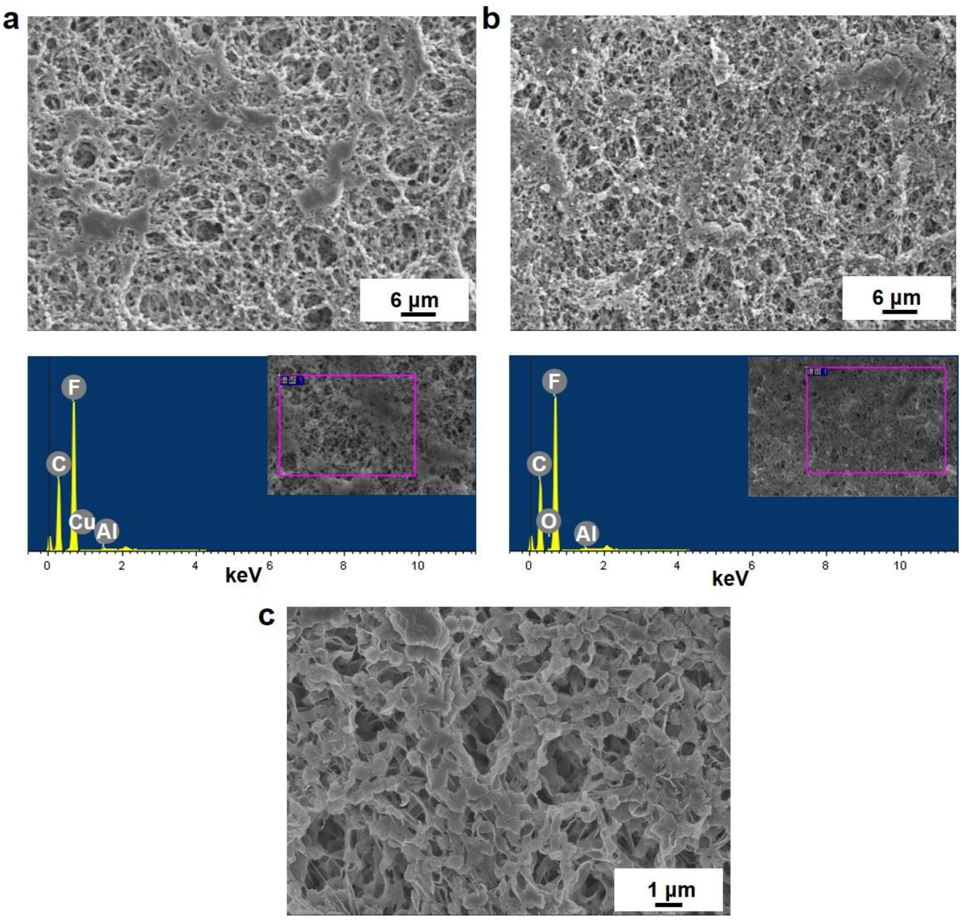

Fig. S8 SEM-EDX analysis for the membranes. SEM images and EDX element analysis results for the membrane **a** before (indicated as pristine) and **b**, **c** after processing the natural seawater for 72 h

Table S2 EDX elemental analysis results for the membrane before (indicated as pristine) and after processing the natural seawater for 72 h

Element	Element mass fraction of different membrane samples (wt.%)	
	Pristine	After SVGMD
C	42.63	41.18
O	\	0.76
F	57.24	57.90
Al	0.14	0.16
Cu	-0.01	\

S11 Water Contact Angle Measurements of Membranes

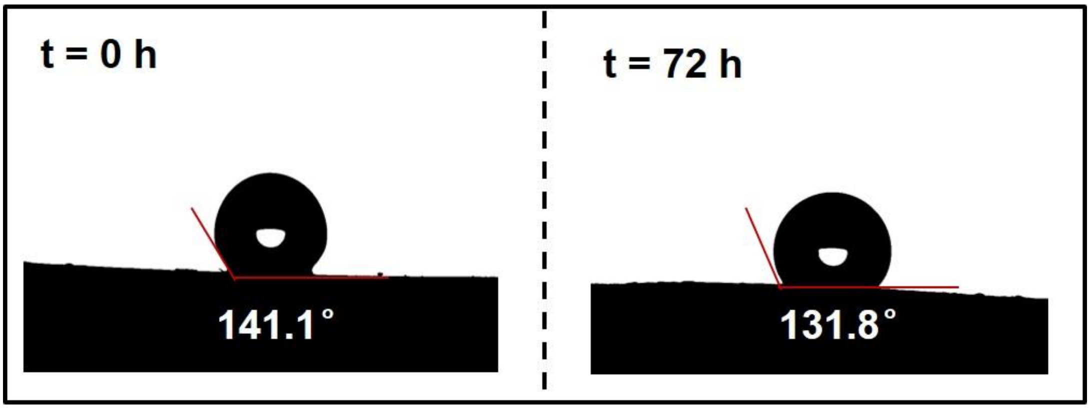

Fig. S9 Water contact angle measurements on the membrane surface. Left: Pristine membrane. Right: After processing mineral oil/seawater mixtures over 72 h

S12 Cyclic Performance Tests of P-G-Ni_{foam}

The salt deposited on the sample would affect the desalination performance [S3]. As shown in the Fig. S10, only a few salt particles deposit on the surface of P-G-Ni_{foam} after processing the natural seawater for 72 h. The long-term stability can be mainly attributed to two factors. Firstly, the surface of G-Ni_{foam} coated with PEDOT-PSS has the characteristics of preventing adhesion of the salt particles to the surface. Secondly, under illumination, the salt ions remained in the P-G-Ni_{foam} result in increased salt concentrations over the natural seawater. Meanwhile, the accumulated ions at the surface of P-G-Ni_{foam} continuously diffuse through the waterways back into the bulk water. The spontaneous ion diffusion driven by salt gradient avoids salt accumulation at the surface of P-G-Ni_{foam}.

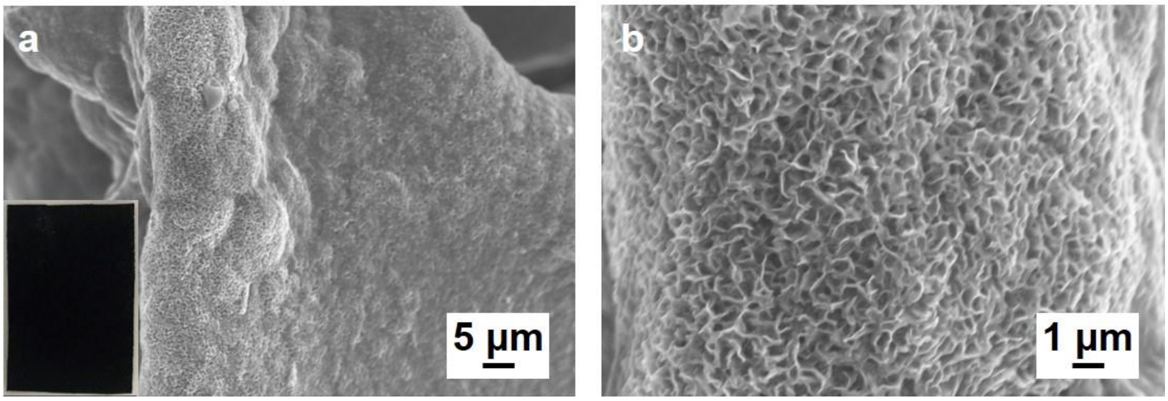

Fig. S10 SEM images of samples after desalination experiments. **a, b** SEM image for P-G-Ni_{foam} after processing the natural seawater for 72 h under solar illumination of 1 kW m^{-2}. Inset: the optical image of P-G-Ni_{foam} after processing the natural seawater for 72 h

S13 Highly-efficient Water Collection

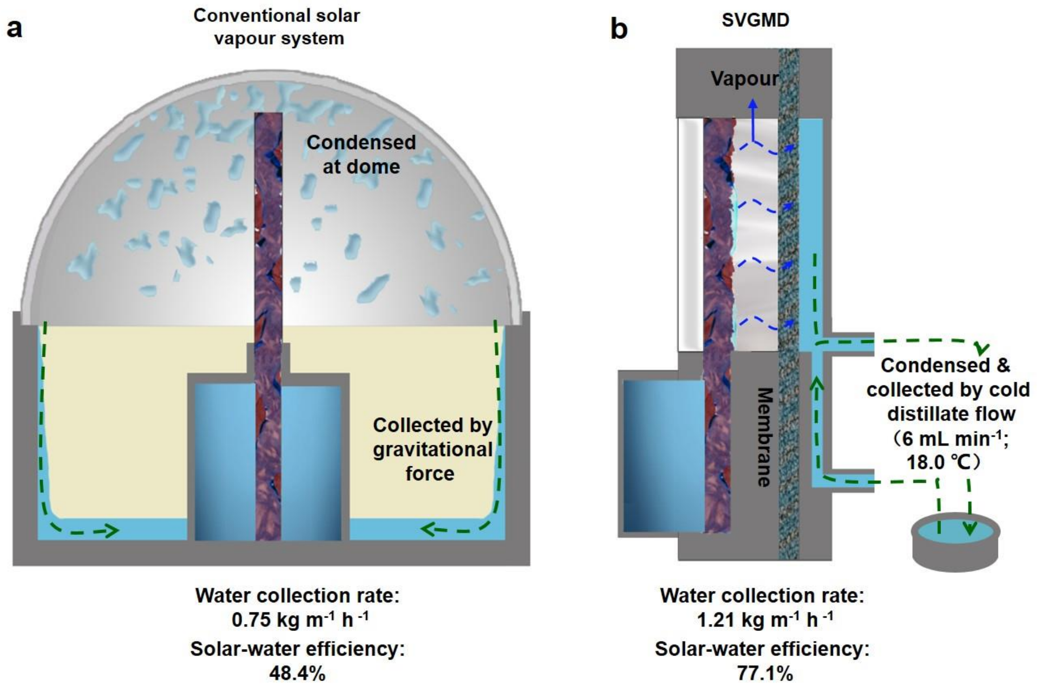

Fig. S11 a Schematic of an apparatus with the water vapour recovered from the glass dome. **b** Schematic of a SVGMD apparatus with the water vapour collected by the distillate flow

S14 Mineral Oil/Seawater Mixture

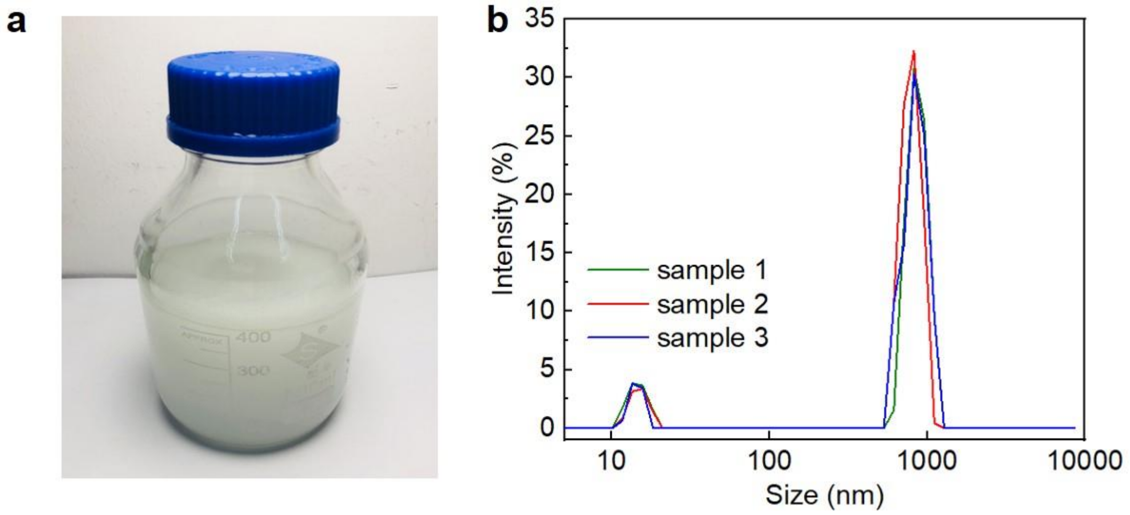

Fig. S12 Mineral oil/seawater mixture used for the experiments with particle size distribution **a** The optical image of the mineral oil/seawater mixture, as one can see stable oil emulsion has been formed. **b** The oil size distribution curve showing oil contents were mostly 0.6 to 1.1 µm in sizes. The oil emulsion was prepared under vigorous stirring for 12 h

Table S3 Composition analysis of seawater from Hainan Island (110.1° E, 20.0° N)

Parameter	Seawater
pH	8.12 ± 0.00
Conductivity (mS cm^{-1})	51.73 ± 0.02
Total dissolved salts (TDS, mg L^{-1})	34,502.41 ± 4.12
Turbidity	0.20 ± 0.00
TOC (mg L^{-1})	10.12 ± 0.92
Water hardness (mg L^{-1} CaCO3 equivalent)	4,461.23 ± 2.09
Sodium (mg L^{-1})	7,334.45 ± 20.78
Potassium (mg L^{-1})	112.32 ± 7.76
Calcium (mg L^{-1})	202.21 ± 9.15
Magnesium (mg L^{-1})	434.23 ± 12.89
Boron (mg L^{-1})	9.51 ± 0.79

S15 Outdoor Purification in the Scaled SVGMD Module Under Natural Sunlight

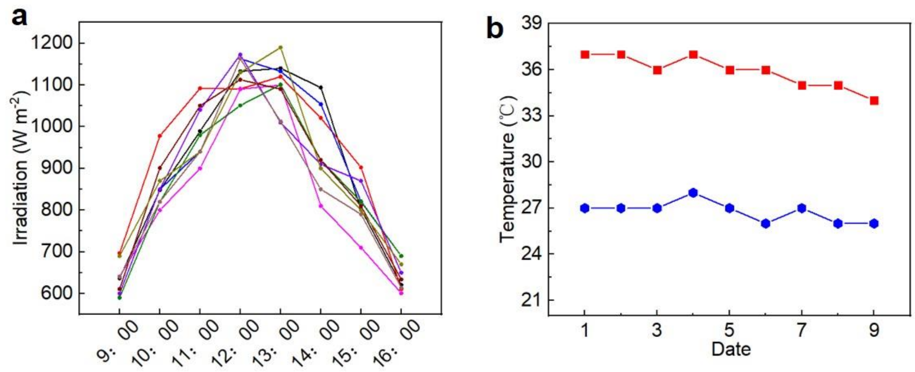

Fig. S13 a Solar radiation recorded over time from 09:00 to 16:00. **b** The temperature change lasted from Aug 5, 2018 to Aug 14, 2018, except August 12 (rainy)

Supplementary References

[S1]Y. Yang, R. Zhao, T. Zhang et al., Graphene-based standalone solar energy converter for water desalination and purification. ACS Nano **12**, 829-835 (2018). https://doi.org/10.1021/acsnano.7b08196

[S2]G. Ni, G. Li, S.V. Boriskina et al., Steam generation under one sun enabled by a floating structure with thermal concentration. Nat. Energy **1**, 16126 (2016). https://doi.org/10.1038/nenergy.2016.126

[S3]H. Ren, M. Tang, B. Guan et al., Hierarchical graphene foam for efficient omnidirectional solar-thermal energy conversion. Adv. Mater. **29**, 1702590 (2017). https://doi.org/10.1002/adma.201702590
